# The Developmental Mechanism of the Root System of Cultivated Terrestrial Watercress

**DOI:** 10.3390/plants12203523

**Published:** 2023-10-10

**Authors:** Jiajun Ran, Qiang Ding, Guangpeng Wang, Yunlou Shen, Zhanyuan Gao, Yue Gao, Xiaoqing Ma, Xilin Hou

**Affiliations:** 1 State Key Laboratory of Crop Genetics & Germplasm Enhancement, Key Laboratory of Biology and Genetic Improvement of Horticultural Crops (East China), Ministry of Agriculture and Rural Affairs of China, Engineering Research Center of Germplasm Enhancement and Utilization of Horticultural Crops, Ministry of Education of China, Nanjing Agricultural University, Nanjing 210095, China; 2020104055@stu.njau.edu.cn (J.R.); 2019204024@njau.edu.cn (Q.D.); 2022204055@stu.njau.edu.cn (G.W.); 2020204027@njau.edu.cn (Y.S.); 2021204025@stu.njau.edu.cn (Z.G.); 2021804158@njau.edu.cn (Y.G.); 2018204021@njau.edu.cn (X.M.); 2 Anhui Jianghuai Horticulture Seeds Co., Ltd., Hefei 230000, China; 3 Nanjing Suman Plasma Engineering Research Institute Co., Ltd., Nanjing 211162, China

**Keywords:** root system, drought, auxin, *DR5::EGFP* watercress, *35S::YUC8* watercress

## Abstract

A well-developed root system is crucial for the rapid growth, asexual reproduction, and adaptation to the drought environments of the watercress. After analyzing the transcriptome of the watercress root system, we found that a high concentration of auxin is key to its adaptation to dry conditions. For the first time, we obtained *DR5::EGFP* watercress, which revealed the dynamic distribution of auxin in watercress root development under drought conditions. Via the application of naphthylphthalamic acid (NPA), 4-biphenylboronic acid (BBO), ethylene (ETH), abscisic acid (ABA), and other factors, we confirmed that auxin has a significant impact on the root development of watercress. Finally, we verified the role of auxin in root development using *35S::NoYUC8* watercress and showed that the synthesis of auxin in the root system mainly depends on the tryptophan, phenylalanine, and tyrosine amino acids (TAA) synthesis pathway. After the level of auxin increases, the root system of the watercress develops toward adaptation to dry environments. The formation of root aerenchyma disrupts the concentration gradient of auxin and is a key factor in the differentiation of lateral root primordia and H cells in watercress.

## 1. Introduction

Watercress (*Nasturtium officinale* R. Br.) is a perennial plant belonging to the Cruciferae family and *Nasturtium* genus. In its natural environment, watercress has a well-developed root system and can quickly occupy growing spaces through creeping or branching, which has led some countries to considering it an invasive species [1]. Watercress grows in water environments such as streams and mountain streams and is known for its roots, which are rich in glucosinolates, flavonoids, and other substances [2]. Its medicinal value has been confirmed in pharmacological experiments [3,4,5,6].

Root is an organ evolved by plants to adapt to land life. It undertakes many functions in the process of plant growth and development, such as support and fixation, absorption and transport, synthesis and transport, storage and reproduction, and so on. Roots have produced rich and varied changes in the process of adapting to the complex and changeable natural environment, which can be roughly divided into four types: main root, fibrous root, adventitious root, and lateral root, but there are also special types of roots, such as parasitic root, aerial root, stem node root, substitute root, and so on. The soil environment where the root system is located is complex. Plants can not only improve their adaptability to stress by producing root secretion via root hairs, but also form a symbiotic relationship with rhizosphere microorganisms [7]. The developed root system can absorb harmful elements in the water, making it an effective water purifier. Root hairs, formed via the differentiation of the root epidermis into H cells [8], are key to water purification by the watercress roots [9,10,11]. They mainly absorb water and nutrients, and their activity is essential for plant growth and development [7]. For example, when plants are subjected to drought or -Pi (phosphate starvation) conditions, they produce a large number of root hairs to increase their absorption area and improve their absorption capacity [12]. Plant hormone is an important part of plant information transmission, and plant hormones often participate in signal transmission by cooperating or antagonizing each other. Auxin signal transduction is very important in the process of root and root hair development. Auxin can be connected to each other via active carbon group (RCS) and ROS signal transduction, and ROS and RCS will regulate auxin signal transduction in a feedforward way to promote root hair elongation [13]. Ethylene also has a great effect on the development of root hair in plant roots. Ethylene can promote the accumulation of reactive oxygen species (ROS) in roots by regulating the activities of respiratory burst oxidase homologues (RBOHC) and peroxidase (*PRX44*) [14]. The increase in the ROS level decreased the expression of the negative regulators of root elongation, *PERK5*, *PERK8* and *PERK13*, and enhanced the expression of positive regulators of root elongation, such as *EXPA7*, *EXPA18* and *COBL9*, thus promoting root hair elongation [15]. The increase in the ethylene content will promote the accumulation of ABA in the root cortex cells, causing the cortical cells to expand and cause the roots to grow laterally. At the same time, ABA can also promote the synthesis and accumulation of auxin in root cortex cells via the *OsbZIP46-OsYUC8* pathway, which will increase the level of auxin and inhibit the elongation of epidermal cells, thus affecting root growth [16].

While there have been many studies on root hair development mechanisms in thale cress (*Arabidopsis thaliana*) [7,17,18,19], there are no studies on the occurrence mechanism of root hairs in watercress. The root system of watercress is developed and grows rapidly. What are the differences in root development between watercress and *Arabidopsis thaliana*? Watercress is fond of water for its growth, and the water culture is mainly used in daily cultivation, which greatly limits the production and popularization of watercress, and root hair is an important structure for watercress to adapt to the arid land environment, purify the water body, and increase yield. it can be regarded as one of the important target characters in watercress breeding. The study on the mechanism of root hair of watercress can lay a foundation for the cultivation, production, and popularization of watercress on land.

## 2. Results

### 2.1. Botanical Characteristics and Tissue Slices of Watercress

**Figure 1:**
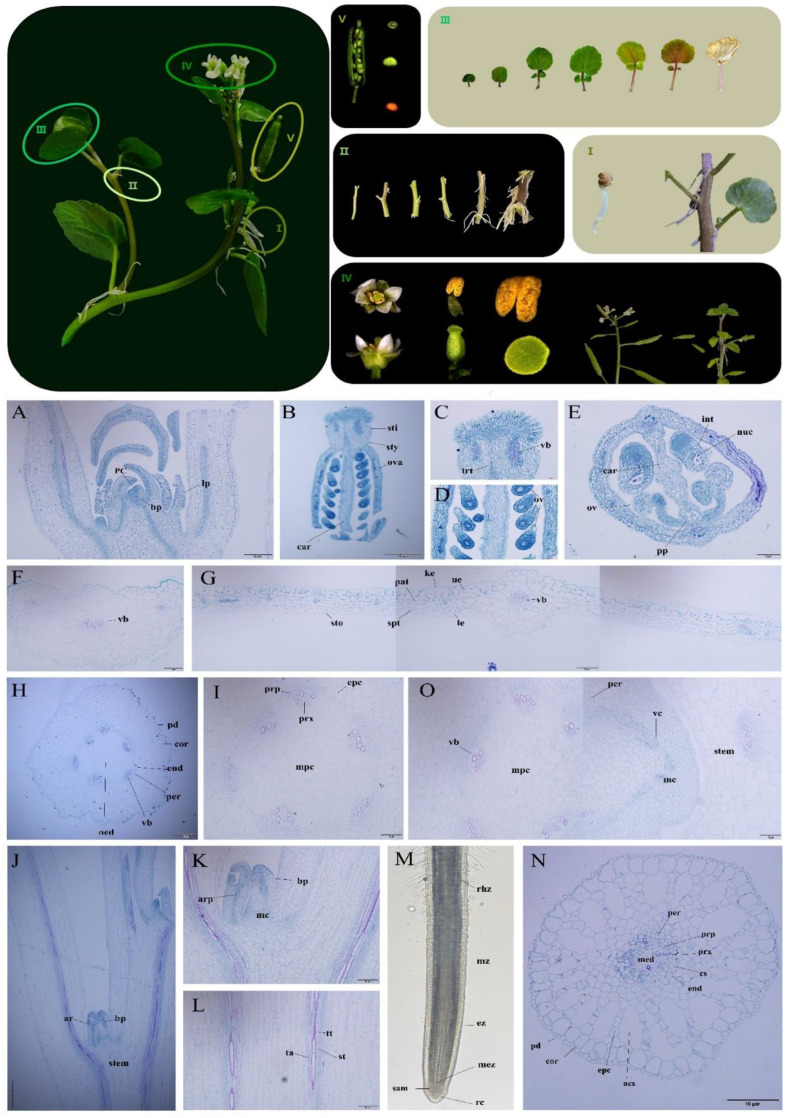
Paraffin sectioning of watercress. (**I**) Two types of watercress roots. (**II**) Watercress stems. (**III**) Watercress leaves. (**IV**) Organ structure and growing points of watercress. (**V**) Development of watercress seeds. (**A**) Growing point. (**B**–**E**) Pistil. (**F**) Cross section of petiole. (**G**) Longitudinal section of petiole. (**H**,**I**) Cross section of stem. (**J**–**L**) Longitudinal section of stem. (**M**) Root. (**N**) Cross section of root. (**O**) Cross section of stem node. **pd** Epidermal cell. **bp** Bud primordium. **lp** Leaf primordium. **mc** Meristematic cell. **vb** Vascular bundle. **pc** Parenchyma cell. **ta** Annular vessel. **tt** Spiral vessel. **st** Sieve tube. **prb** Primary root primordium. **ar** Adventitious root. **vc** Vascular cambium. **sty** Style. **sti** Stigma. **pi** Pistil. **ova** Ovary. **int** Integument. **nuc** Nucellus. **car** Carpellary wall. **ov** Ovule. **trt** Central transmitting tissue. **pp** Parietal placentation. **sto** Stoma. **spt** Spongy tissue. **pat** Palisade tissue. **ke** Cuticle. **ue** Upper epidermis. **le** Lower epidermis. **cor** Cortex. **end** Endodermis. **epc** Epidermal cell of cortex. **per** Pericycle. **med** Medulla. **mpc** Medullary parenchyma cell. **prp** Primary phloem. **prx** Primary xylem. **stem** Stem node. **rhz** Root hair zone. **mz** Maturation zone. **ez** Elongation zone. **mez** Meristematic zone. **rc** Root cap. **sam** Shoot apical meristem. **cs** Casparian strip. **acs** Air cavity structure.

We tracked and recorded the growth and development process of watercress and summarized its growth and structural characteristics (Figure 1). The roots of watercress are divided into two types. The first type is the primary root formed by the embryonic root, which exists in the seedling stage and withers during the nutritional growth period, losing its function. The second type is the adventitious root produced at the leaf axil, which has a short and robust germination time. Watercress grows in a semi-prostrate and semi-upright state. The upright part of the plant has few root hairs, but when the humidity is low, root hairs grow in large numbers (Figure 1I). Root paraffin sections show that the endodermis of the root has a well-developed Casparian strip and a well-developed aerenchyma structure after maturity (Figure 1N,M). In the seedling stage, the stem is light green, round in the cross section, and easy to break. As the plant grows, its color gradually changes to purple, and the cross section gradually becomes polygonal. With the increase in fiber content, the toughness of the stem increases, making it less prone to breakage (Figure 1II). Cross-sectional slices of the stem show well-developed vascular bundles and a clearly visible medullary structure. The cambium layer at the node expands outward while the central cylinder cells differentiate to produce roots and buds (Figure 1H–L,O). The growing point is formed via the leaf primordium wrapping around the bud primordium (Figure 1A).

The leaves of this plant are odd-pinnately compound leaves, and the leaf stalk base partially encircles the stem segment. During the seedling stage, the leaves resemble bean pods, are deep green, and are not compounded. During the nutritional growth period, the leaf blades expand into a nearly circular shape, turn green, and produce one or more pairs of leaves (the number of leaves varies with the variety). During the senescence period, the leaves rapidly wither and turn yellow, while the leaf stalk changes from green to purple (Figure 1III). The transverse section of the leaf blade and leaf stalk shows that the leaf blade is thin, with the sponge tissue and palisade tissue not clearly distinguishable. The upper epidermal cells are relatively large and tightly arranged, and the leaf stalk contains more vascular bundles (Figure 1F,G). The flower is small, bisexual, and has typical characteristics of the Brassicaceae family. It has four white petals arranged in a cross shape, four green sepals, tetradynamous stamens, and a bottle-shaped pistil with the ovary superior and many ovules. The style is nearly circular, and the papillate structure is not obvious (Figure 1B–E). At the beginning of the flowering period, many flowers gather into an umbel-like inflorescence. During the flowering process, the axis elongates to form a racemose inflorescence. At the end of the flowering period, the pistil style degenerates, the corolla and sepals wither, and the naked seed pod cracks easily after maturation. During seed maturation, the volume gradually increases, and the seed coat gradually hardens and turns yellow from green. When the bean pod vegetable is in a high-humidity and high-nutrition environment, the apical primordium differentiates to form a bud primordium and continues to grow (Figure 1IV).

### 2.2. Transcriptome Analysis of Root Tissue in Watercress

**Figure 2:**
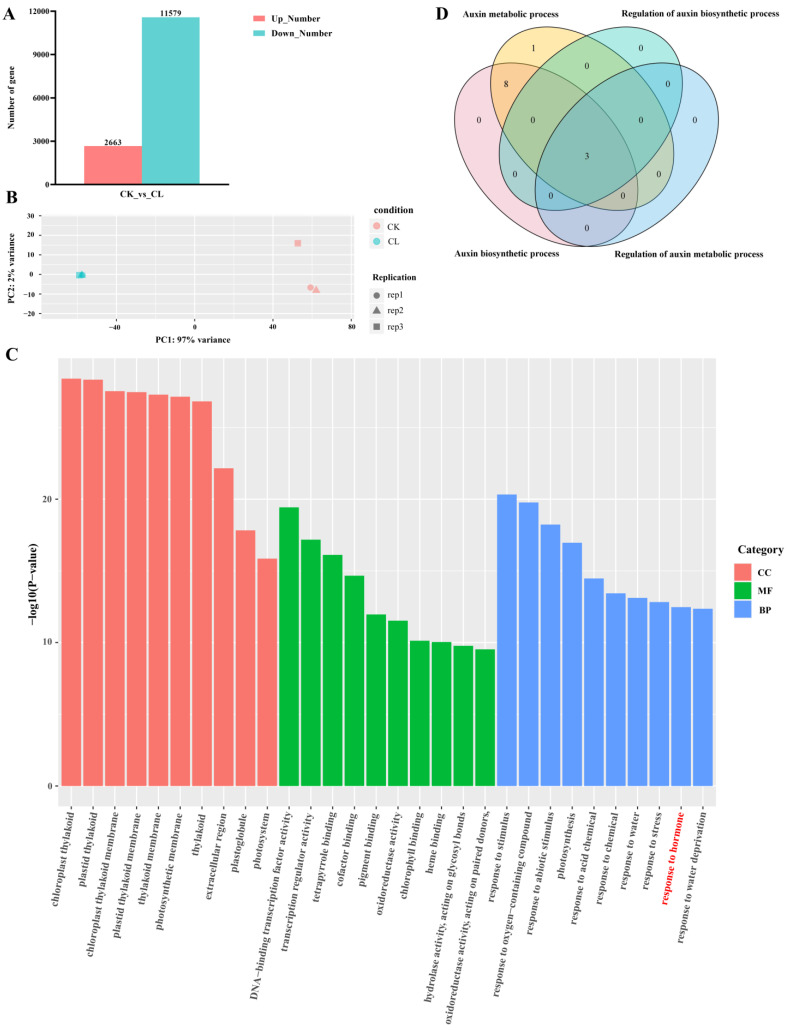
Transcriptome analysis of root. (**A**) A number of DEGs in the transcriptome. (**B**) PCA analysis for each sample in the transcriptome. (**C**) GO enrichment analysis of differentially up-regulated genes. (**D**) Venn diagram of the genes associated with GO terms related to auxin metabolism.

We performed RNA-seq on the root samples collected before and after root hair development and found significant differences in the gene expression. There were 14,242 differentially expressed genes (DEGs) (|log2FoldChange| > 1, *p*-value < 0.05), with 2663 genes up-regulated and 11,579 genes down-regulated during root hair development (Figure 2A). Principal component analysis (PCA) showed that there was no difference in the biological replicates (Figure 2B). To identify important biological pathways during root hair development, we conducted gene ontology (GO) enrichment analysis on the differentially expressed genes and found the term “response to hormone stimulus” enriched in the up-regulated genes, which included 304 genes (red texts in Figure 2C). By examining the annotation results of the transcripts (Tables S1 and S2), we found that among these genes, those involved in auxin synthesis were the most numerous. Auxin is known to be a regulator of many agronomic traits, including root development, tillering, and drought resistance, which directly affects the crop yield and quality [20]. Therefore, we speculate that auxin plays an important role in the root development of watercress. We further analyzed the GO enrichment results and found four terms related to auxin synthesis (Tables S1 and S2). We extracted gene ids from these four terms for the Veen map and found that these four terms jointly contained three genes: *NoPIF4*, *NoPIF5,* and *NoYUC8* (Figure 2D). The enzymes and genes related to auxin synthesis are mostly dependent on the tryptophan pathway, including the indole-3-acetaldoxime (IAOx) pathway, tryptamine pathway, indole-3-acetamide (IAM) pathway, and indole-3-pyruvic acid (IPA) pathway. Among them, the IPA pathway is the most important pathway for auxin biosynthesis in plants [21]. This pathway mainly involves two steps mediated by TAA and YUC to promote auxin synthesis [22,23,24]. Via the analysis of differential gene expression, we identified the up-regulated genes, *NoYUC1*, *NoYUC3,* and *NoYUC8*, and the down-regulated gene, *NoYUC6*, but did not find any annotated genes in the aminotransferase TAA family (Tables S1 and S2). Thus, based on these findings, we hypothesize that in the development of watercress roots, the synthesis of auxin may depend on the YUC-mediated reaction, with the *NoYUC8* gene playing a key role in auxin synthesis.

### 2.3. Dynamic Distribution of Auxin in Root Development of Watercress

**Figure 3:**
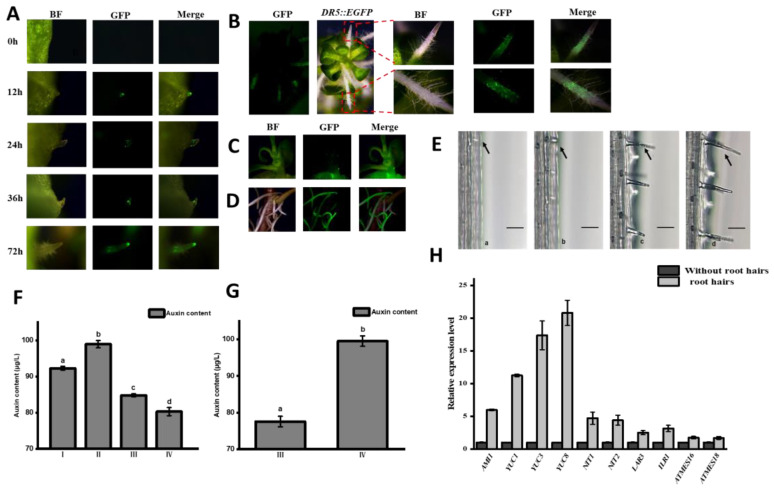
GFP fluorescence, auxin content, and related gene expression level of *DR5::EGFP* watercress. (**A**) Changes in GFP fluorescence during root development of *DR5::EGFP* watercress. (**B**) GFP fluorescence of *DR5::EGFP* watercress roots under drought stress. (**C**) *DR5::EGFP* watercress. (**D**) GFP fluorescence of *DR5::EGFP* watercress under hydroponic conditions. (**E**) Root hair development of watercress. (**F**) Auxin content during the four stages of root growth. (**G**) Changes in auxin content in the maturation zone before and after root hair formation. (**H**) Expression levels of auxin-related genes before and after root hair formation. All the bars indicate mean ± SD. Letters indicate significant difference analysis (*p* < 0.05, Student’s *t*-test).

*DR5::EGFP* transgenic watercress was cultured in a Murashige and Skoog (MS) medium containing 0.2 M of PEG to simulate a drought environment, and the GFP fluorescence signal of the *DR5::EGFP* watercress root development was tracked and observed. It was found that there was no GFP fluorescence signal when the root primordium cells were not formed in the central cylinder cells of the stem, but at 12h, when the root primordium emerged through the epidermal cells of the stem to form a young root, the GFP fluorescence signal in the root tip meristem was strong; after 24 h of young root growth, the GFP fluorescence signal in the root tip meristem weakened, and the GFP fluorescence signal appeared at the edge of the elongation zone; at 36 h of root growth, the GFP fluorescence signal in the root tip meristem was weak, but some were concentrated in the root meristem; and at 72 h, when the root hair sprouted in the root maturation zone, the GFP fluorescence signal was still weak but distributed in the root hair zone (Figure 3A). Root hair development has four stages: first, the differentiation of the root epidermal cells to form the H cell (a); second, the H cell forms a hair-like structure (b); third, it protrudes and elongates to form the rudiment of the root hair (c); finally, the root hair develops and matures (d). Drought treatment inhibits the growth of watercress and promotes the growth of a large number of root hairs in the upright stem. The root length becomes shorter, and a large amount of GFP fluorescence signal is concentrated in the root hair area (Figure 3B). When watercress is grown in a hydroponic environment, the upright part of the root continues to elongate, and the entire root has a strong GFP fluorescence signal during elongation (Figure 3C). These findings indicate that auxin is involved in the entire process of root and root hair development in watercress.

We measured the content of auxin in roots at four stages: (I): before the root primordium emergence through the epidermis, (II): after the root primordium emergence through the epidermis, (III): during root elongation, and (IV): root maturation (Figure 1H). We found significant differences in the concentration of auxin at each stage. When the auxin content in the stem node epidermis increased, the root primordium undergoing differentiation from the central cylinder formed a young root. As the auxin concentration continued to decrease, the young root gradually developed and matured, indicating that low concentrations of auxin could promote root elongation and development. We also measured the expression levels of key genes involved in the genes of the auxin tryptophan synthesis pathway (*NoAM1*, *NoYUC1*, *NoYUC3*, *NoNIT1,* and *NoNT2*) and the genes involved in auxin conjugation and release (*NoLAR3*, *NoILR1*, *NoATMES16,* and *NoATMES18*) in the maturation zone at stages III and IV (Figure 1G). We found that the expression levels of the key genes (*NoAM1*, *NoYUC1*, *NoYUC3*, *NoNIT1,* and *NoNT2*) in the auxin tryptophan synthesis pathway were significantly increased before and after the formation of root hairs, especially *NoYUC1* and *NoYUC3*, while the expression levels of the genes involved in the amino acid complex pathway (*NoLAR3* and *NoILR1*) and the methylation pathway (*NoATMES16* and *NoATMES18*) were slightly increased. This suggests that changes in auxin concentration in the roots are mainly caused by the de novo synthesis of auxin rather than the conjugation and release of auxin. Based on the changes in the GFP fluorescence, the auxin content, and the expression levels of genes involved in auxin metabolism during root growth and development, we speculate that auxin is an important influencing factor in the formation of the primary root and root hairs in watercress.

### 2.4. Other Important Factors Affecting the Occurrence of Root Hairs in Watercress

**Figure 4:**
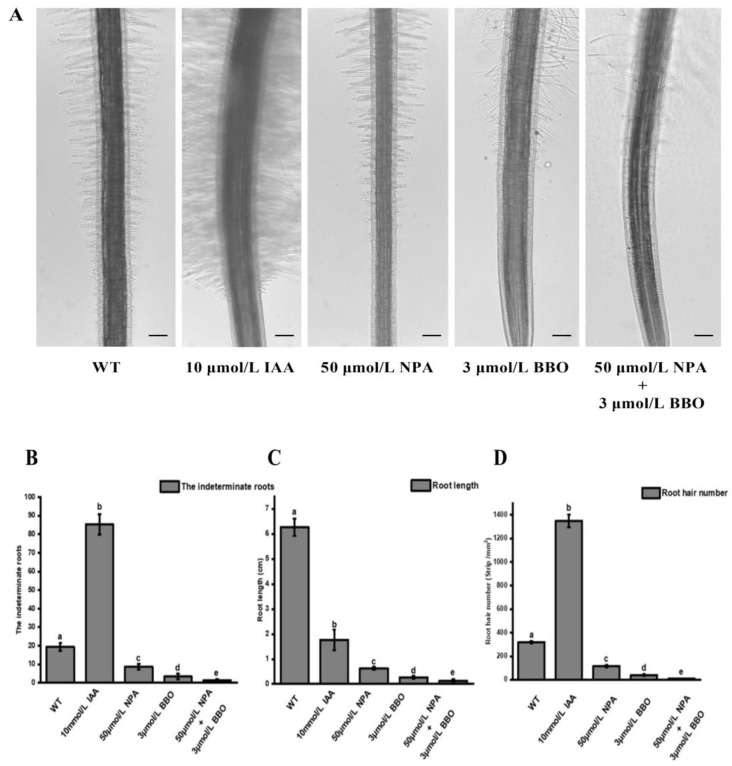

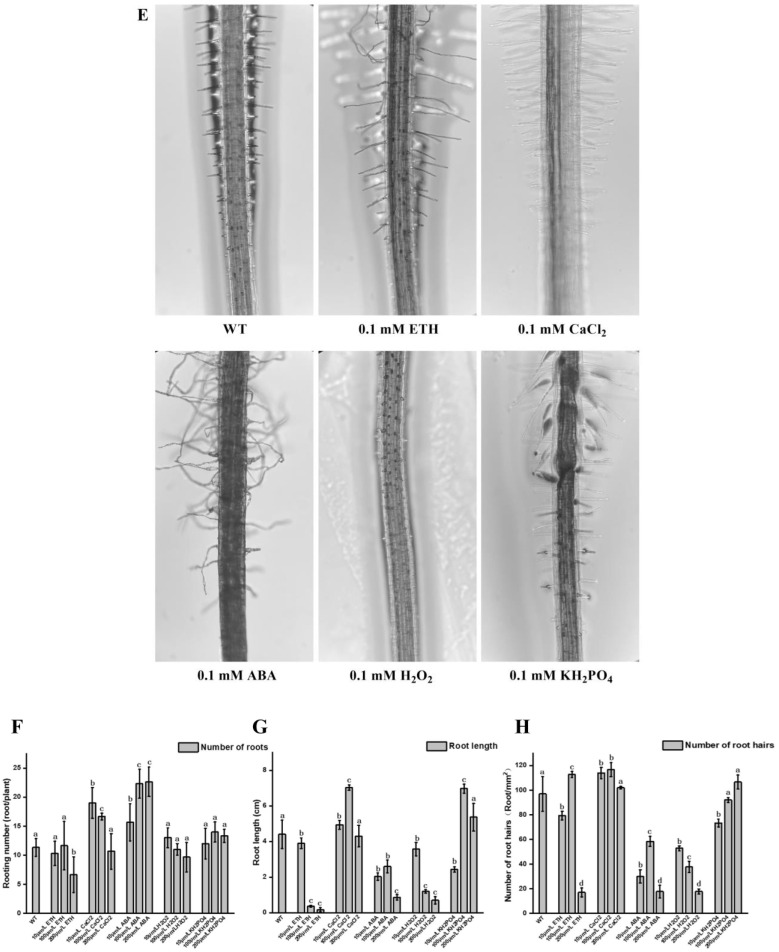
Effects of different treatments on the development of watercress roots. (**A**) Effects of IAA, BBO, and NPA on root system development. (**B**) Effects of IAA, BBO, and NPA on root formation. (**C**) Effects of IAA, BBO, and NPA on root length. (**D**) Effects of IAA, BBO, and NPA on root hair development. (**E**) Effects of ETH, CaCl_2_, ABA, H_2_O_2_, and KH_2_PO_4_ on root system development. (**F**) Effects of ETH, CaCl_2_, ABA, H_2_O_2_, and KH_2_PO_4_ on root formation. (**G**) Effects of ETH, CaCl_2_, ABA, H_2_O_2_, and KH_2_PO_4_ on root length. (**H**) Effects of ETH, CaCl_2_, ABA, H_2_O_2_, and KH_2_PO_4_ on root hair development. All the bars indicate mean ± SD. Letters indicate significant difference analysis (*p* < 0.05, Student’s *t*-test).

In order to verify the specific effect of auxin on the root hair formation of watercress, watercress was treated with IAA, NPA, BBO, and NPA+BBO for 15 days, and then the changes in the rooting number, root length, and root hair number were counted. The results showed that the exogenous application of IAA could promote the formation of watercress roots and root hairs but inhibit root elongation. After the application of the auxin transport inhibitor NPA, the number of roots and root hair quantity was significantly lower. The effect of the auxin synthesis inhibitor BBO was more significant. Although the number of roots, root length, and root hair number were significantly reduced after the co-application of NPA and BBO, some root hairs still occurred (Figure 4A–D). This indicates that auxin is an important factor influencing the development of roots and root hairs in watercress. Once the synthesis or transport of auxin is inhibited, not only the root primordium and H cells are inhibited, but also the number of roots and root hairs are reduced, and root elongation is also inhibited. However, even when both the synthesis and transport of auxin are blocked, some root hairs still occur, indicating that there are other pathways besides auxin that can affect the development of root hairs in watercress.

In order to explore the effect of other factors on the root development of watercress, watercress was treated with ETH, CaCl_2_, ABA, H_2_O_2_, and KH_2_PO_4_. After 15 days, the number of root, root hair, and root length were measured. The results showed that ABA and a low concentration Ca^2+^ could promote the development of root primordia and increase the number of roots, while a high concentration of ETH and MNT could inhibit the development of root primordia and reduce the number of roots. An amount of 0.1 mM Ca^2+^ and KH_2_PO_4_ could promote root elongation, while ETH, MNT, reactive oxygen species, and a high concentration of Ca^2+^ could inhibit root elongation. A total of 0.1 mM ETH and 0.1 mM Ca^2+^ could promote root hair development, while ABA, reactive oxygen species, and a high concentration of ETH could inhibit root hair development (Figure 4E–H). The indole-3-acetic acid-auxin response factor (*IAA-ARF*) is an important signal module in plant lateral root development [25]. In *Arabidopsis thaliana*, *IAA14* and *IAA18* can regulate lateral root formation via the interaction with *ARF7* and *ARF19* [26,27], while *IAA19* can regulate not only lateral root formation but also Hypocotyl growth via the interaction with *ARF7* [28]. The indole-3-acetic acid-auxin response factor (*IAA-ARF*) auxin signal module formed by lateral roots has been proven to play a key role in auxin-induced callus formation [29,30]. Under normal growth conditions, CaM-IQM (calmodulin IQ motif) in the middle column sheath interacts with IAA protein to release ARF activity after sensing a low level of the Ca^2+^ gradient. The low level Ca^2+^ signal is coordinated with the endogenous auxin signal, which regulates lateral root initiation. When CaM-IQM senses a high level of the Ca^2+^ gradient, it releases IAA and inhibits ARF activity. It cooperates with auxin-induced IAA proteolysis to activate the root development program ectopic, thus regulating the formation of callus [31]. These findings suggest that ETH, CaCl_2_, ABA, H_2_O_2_, and KH_2_PO_4_ have clear dose-dependent effects on the development of root primordia, root elongation, and the H cells of watercress, but compared to auxin, they are not the main factors affecting the development of roots and root hairs.

### 2.5. Regulation of NoYUC8 on Root Development in Watercress

**Figure 5:**
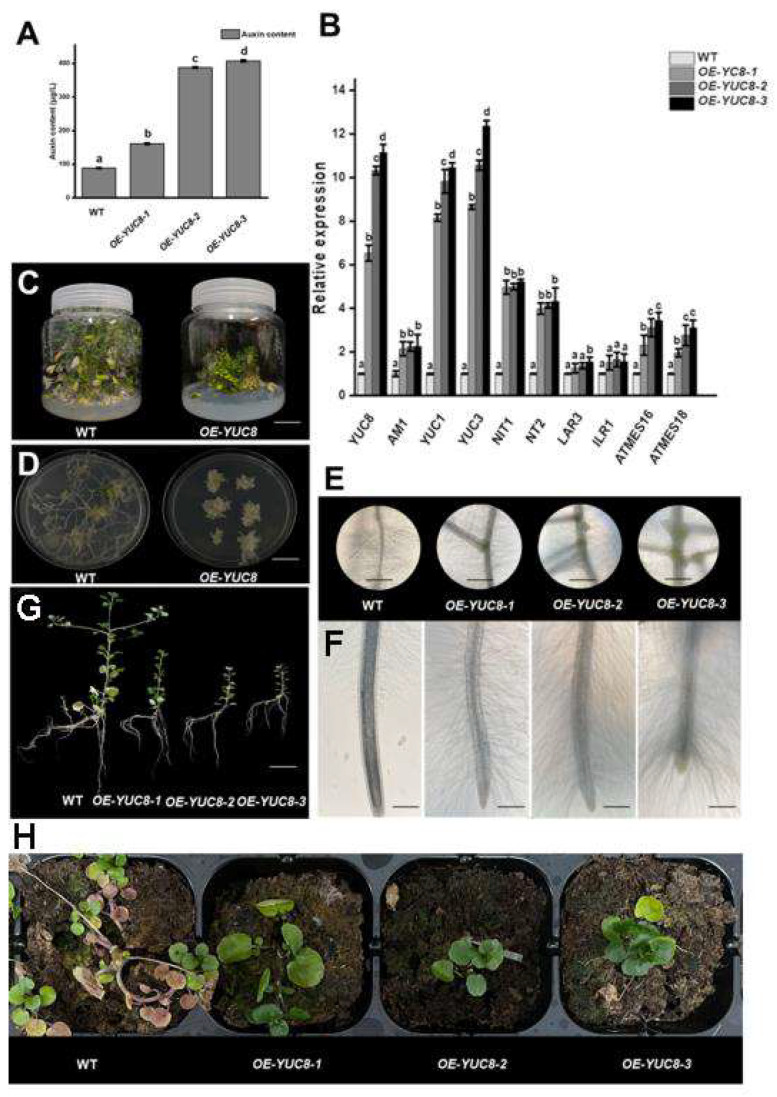
The effect of *NoYUC8* overexpression on watercress growth and development. (**A**) The effect of *NoYUC8* overexpression on watercress growth hormone. (**B**) The effect of *NoYUC8* overexpression on gene expression in the auxin synthesis pathway. (**D**,**F**) The effect of *NoYUC8* overexpression on watercress development. (**C**,**E**) The effect of *NoYUC8* overexpression on watercress root development. (**G**) The effect of *NoYUC8* overexpression on watercress growth in aquatic environments. (**H**) The effect of *NoYUC8* overexpression on watercress growth under drought conditions. All the bars indicate mean ± SD. Letters indicate significant difference analysis (*p* < 0.05, Student’s *t*-test).

During the regeneration stage, the *35S::NoYUC8* watercress plants showed significant dwarfing (Figure 5D). To better understand the effect of *NoYUC8* on root development in watercress, we removed the shoot of the wild-type and *35S::NoYUC8* watercress, and the stem segments were cut and inoculated on an MS medium for 15 days in order to observe the root changes later. We found that the stem segments of the *35S::NoYUC8* watercress produced more roots and lateral roots, shorter root lengths, and more root hairs (Figure 5C,E). We measured the expression levels of the *NoYUC8* gene in the OE-NoYUC8-1, OE-NoYUC8-2, and OE-NoYUC8-3 strains and found that the expression level of *NoYUC8* in OE-NoYUC8-1 increased by six times, while in OE-NoYUC8-2 and OE-NoYUC8-3, the expression level of *NoYUC8* increased by 10–11 times. After measuring the expression levels of auxin synthesis-related genes and the auxin concentration, it was found that in the three OE-NoYUC8 strains, the expression levels of auxin synthesis-related genes were all increased, and the expression levels of the homologous genes of *NoYUC8* in the tryptophan synthesis pathway, *NoYUC1*, and *NoYUC3*, increased most significantly (Figure 5B). The content of auxin in the OE-NoYUC8-2 and OE-NoYUC8-3 strains was about four times higher, and the content of auxin in the OE-NoYUC8 strain was about two times higher than that in the wild-type (Figure 5A). Later, the three *35S::NoYUC8* watercress strains were placed with the wild-type on a 1.4% agar medium for 7 days, and observation under a microscope showed that with the increase in the *NoYUC8* expression level, as the lateral roots increased, the boundary between the root elongation zone and the maturation zone became less distinct, and the root hairs became longer and denser (Figure 5C). This indicates that the overexpression of *NoYUC8* enhances the expression of auxin synthesis-related genes, such as YUCs and NITs, which increases the level of auxin in watercress, inhibits its growth, and at the same time, promotes the development of roots and root hairs.

To investigate the effect of *NoYUC8* on the production and cultivation of watercress, we cultured three different strains of watercress, including wild-type and *35S::NoYUC8* watercress, in a Yamazaki nutrient solution and dry substrate (watered only during planting) for 15 days. Under hydroponic conditions, both the wild-type and *35S::NoYUC8* watercress exhibited semi-erect and semi-prostrate growth, but the wild-type watercress had longer internodes, taller plant height, more lateral branch sprouts, and longer roots, while the *35S::NoYUC8* watercress showed shorter internode spacing, dwarfed plant height, more root development, and shorter roots (Figure 5G). In the dry substrate cultivation, the growing point and base leaves of the wild-type watercress withered and grew prostrate, with more lateral branches extending outward in search of water, while all three strains of the *NoYUC8* watercress maintained relative erectness, with relatively green growing points and leaves, no lateral branches, and a more visibly healthy growth status than the wild-type watercress (Figure 5H). This indicates that the overexpression of *NoYUC8* can enhance the adaptability of watercress to drought conditions, which is beneficial to the growth of watercress in arid environments.

The above results indicate that the expression levels of auxin synthesis genes *NoYUC8*, *NoYUC1*, and *NoYUC3*, as well as their homologs, were increased, resulting in elevated levels of auxin in the plant body of watercress. This, in turn, induced the de-differentiation of the central cylinder cells at the stem–node junction to form root primordia cells. During the root elongation stage, the auxin concentration gradient was disrupted, polar elongation was obstructed, and abnormal root elongation occurred, resulting in no significant difference between the root elongation zone and the maturation zone. As the auxin level increased in the root maturation zone, it not only promoted the de-differentiation of the pericycle cells to form lateral root primordia, thus promoting lateral root initiation, but it also promoted the elongation of root hairs while promoting more epidermal cells to differentiate into H cells.

## 3. Discussion

Root activity is essential for plant growth and development, and as an important part of the root system, root hairs can both increase the absorption area of roots and enhance their ability to absorb water and nutrients. They also interact with the rhizosphere microbial community, regulating the root environment [12]. Root development is closely related to plant hormones, with many studies focusing on the effects of auxin (IAA), ethylene (ETH), abscisic acid (ABA), and others on root development. Local fluctuations in auxin can induce the differentiation of pericycle cells into root primordial cells, which then undergo a series of asymmetric cell divisions to ultimately form root primordia [32]. The auxin synthesized in the quiescent center region is transported towards the base through the epidermis, refluxes through the central cylinder, forms a concentration gradient, and triggers polar root growth, until it breaks through the epidermis to form roots [33,34,35]. Based on the tissue sections of watercress and the changes in the *DR5::EGFP* fluorescence signals, we assume that local fluctuations in auxin occurred at the stem–node junction of watercress, inducing the differentiation of central cylinder cells into the root primordial. Root primordia developed via asymmetric cell division and broke through the epidermis to form young roots. The quiescent center region of young roots continued to synthesize auxin, which was transported towards the root base through the epidermal cells, refluxed through the central cylinder, formed a concentration gradient, and induced polar root growth. Once the root matures, a large number of cortical cells die, forming well-developed aerenchyma structures. Auxin reflux through the central cylinder was obstructed, and it accumulated in the epidermis of the root maturation zone, inducing the differentiation of epidermal cells into H cells, which produce root hairs and matured under a high concentration of auxin. The main reason for the limited production of lateral roots in mature watercress roots is assume to be due to the formation of an aerenchyma structure, which hinders the reflux of auxin through the epidermal cells into the central cylinder, thereby reducing the auxin level in the central cylinder and making it unable to induce the de-differentiation of central cylinder cells into lateral root primordia.

Studies on the mechanism of root hair development in thale cress are relatively thorough. Root epidermal cells can be divided into H cells and N cells. H cells develop into root hairs [7], while the transcriptional complex WER-GL3/EGL3-TTG induces the expression of *CPC* and the negative regulatory factors of root hair—*GL2*, which determines the differentiation of epidermal cells into N cells [36]. *CPC* in N cells can transfer to adjacent epidermal cells and compete with WER for binding to the GL3/EGL3-TTG complex, thus suppressing the *GL2* expression and promoting differentiation into H cells [37,38]. Actin filaments and auxin are involved in the formation of root hairs in H cells [39,40]. Auxin and bHLH transcription factor *RHD6* can regulate the expression of genes related to physiological activities such as cell wall modification, membrane transport, ion transport, and cytoskeleton in root hair cells by controlling the expression of *RLS2* and *RLS4*, thereby regulating root hair elongation [38,41,42]. K^+^ and Ca^2+^ have been proven to be necessary for root hair elongation [43,44], and ROS is a key signal in the complex network of root hair elongation [45]. In rice roots, after soil compaction, ETH diffusion is obstructed, and a local increase in the ETH concentration activates *OsEIL1*. *OsEIL1* directly enhances the expression of *NoYUC8* and promotes ABA synthesis to increase the ABA content in the root, thereby activating *OsbZIP46*. *OsbZIP46* enhances the *NoYUC8* expression, leading to an increase in the level of root auxin, ultimately inhibiting cell elongation in the root elongation zone and promoting the lateral growth of cortical cells, resulting in short and stubby roots [46].

The watercress has a well-developed root system and grows rapidly, making it a key crop for dryland cultivation and a good material for studying root development. However, there is currently no research on root and root hair development in watercress. Via transcriptome analysis of the watercress root system, we found that the expression of genes related to auxin increased significantly before and after root hair formation. Therefore, we constructed a genetic transformation system and obtained *DR5::EGFP* watercress for the first time. Based on changes in the GFP fluorescence signal, we revealed, for the first time, the dynamic changes of auxin during the development of the watercress root system, with local changes in the auxin concentration in the root mainly achieved via the TAM pathway of tryptophan synthesis. However, we could not confirm whether auxin is the main influencing factor. Therefore, we stimulated watercress with common root development factors such as K^+^, Ca^2+^, Pi, ABA, ETH, and reactive oxygen species. Finally, we confirmed that the high levels of auxin locally are critical for the formation of root primordia and H cells in watercress, while the low levels of auxin are necessary for root elongation. Then, we obtained *35S::NoYUC8* watercress for the first time via a genetic transformation system and found that the expression of *NoYUC8* and homologous genes increases the level of auxin, promoting the differentiation of root primordia and H cells. High levels of auxin also inhibit cell elongation in the elongation zone and promote the lateral growth of cells, resulting in no clear boundary between the elongation zone and the maturation zone.

## 4. Materials and Methods

### 4.1. Plant Materials, Growth Conditions, and Treatments

The watercress used in this study is a sequencing material provided by Nanjing Agricultural University (relevant information has not been released yet). The watercress was cultured in Hogan’s solution under the conditions of light for 16 h and dark for 8 h, with the temperature at 24 °C.

Watercress with the same growth state and no root was inoculated into agarose containing 10 μmol/L of IAA, 50 μmol/L of NPA, 3 μmol/L of BBO and 50 μmol/L of NPA + 3 μmol/L of BBO for 15 days.

In addition, ETH, CaCl_2_, ABA, H_2_O_2_, and KH_2_PO_4_ were added to 1.4% agarose; 0.01 mM, 0.1 mM, and 0.2 mM^3^ concentration gradients were set in each treatment; and watercress with a consistent growth state and no roots were inoculated on it and cultured for 15 days.

### 4.2. Paraffin Sectioning of Watercress

The roots, stems, leaves, and flowers of watercress were photographed to record their growth and developmental changes. The samples were then fixed in FAA (50%) for 12 h, followed by washing, dehydration, and other steps to prepare the paraffin sections. Inverted microscopy was used to observe the samples.

### 4.3. Auxin Determination

Watercress was cultured in agar blocks. When the roots began to sprout, 0.1 g of fresh stem epidermis samples were collected at stage (I): before the root primordium emergence through the epidermis, and stage (II): after the root primordium emergence through the epidermis, respectively. An total of 0.1 g of fresh roots were collected at stage (III): during root elongation, and stage (IV): root maturation, respectively. The maturation zone of root hairs at stage III and stage IV were collected, weighing 0.1 g, respectively. In addition, 0.1 g of both transgenic watercress with *35S::NoYUC8* and wild-type watercress fresh roots, respectively, were also collected. The fresh samples were frozen, and their auxin content was determined using the indole-3-acetic acid (IAA) ELISA kit (brand: MEIMIAN, Jiangsu Meimian Industrial Co., Ltd., Yancheng, China).

### 4.4. Vectors and Identification of Transgenic Watercress

Details of the primers used can be found in the Table S1. PCR were used to detect 35S::*NoYUC8*, and the *DR5::EGFP* watercress can be found in the Figure S1.

### 4.5. Measurement of Root Length and Root Hair Number in Watercress

Root length was measured using an electronic caliper, and the number of root hairs in the maturation zone was counted under the same field of view using photographs.

### 4.6. Genetic Transformation of Watercress

For details on the genetic transformation of watercress, please refer to end of the Supplementary Material S1.

### 4.7. Transcriptome Sequencing of Watercress Adventitious Roots at Different Stages

A total of 0.2 g fresh samples of the maturation zone without root hairs in stage III and 0.2 g fresh samples of the root hair area with formed root hairs in stage IV were collected for transcriptome sequencing. RNA extraction, purification, and library construction were conducted, and the resulting libraries were subjected to paired-end (PE) sequencing using next-generation sequencing (NGS) based on the Illumina sequencing platform. The raw data were first filtered by removing the low-quality reads with an average quality score below Q20, the reads with adapters, and the reads shorter than 50 bp. The high-quality reads were then assembled into clusters, and the longest transcript in each cluster was selected as a Unigene. The Unigenes were subsequently annotated for GO, KEGG, eggNOG, SwissProt, Pfam, and other functional categories. Additionally, the filtered reads were mapped to the Unigenes to obtain the Reads Count of each Unigene, and further analyses were performed, including differential expression analysis and enrichment analysis of the samples.

## Data Availability

Some or all data generated or used during the study are available from the corresponding author on reasonable request.

## Supplementary Materials

The following supporting information can be downloaded at: https://www.mdpi.com/article/10.3390/plants12203523/s1, Figure S1: The PCR test results of 35::NoYUC8 (A) and DR5::EGFP (B) transgenic watercress; Figure S2: Effect of NoYUC8 gene overexpression on watercress cultivated on land; Figure S3: Effect of NoYUC8 gene overexpression on watercress; Table S1: Primers of auxin related genes; Table S2: GO enrichment of transcriptome DEGs; Table S3: Gene annotation.
