# Supplementary material for: The Developmental Mechanism of the Root System of Cultivated Terrestrial Watercress
Source: Plants (Basel). 2023 Oct 10;12(20):3523. doi: 10.3390/plants12203523 (PMC10610301; doi:10.3390/plants12203523)
Supplement: Supplementary file 1 [file plants-12-03523-s001.zip › Supplementary material 1.pdf]

Table S1 Primers of auxin related genes

Gene Name	Primer sequence
DR5-F	AGGGGATCCATGGCTCCAAAGAA
DR5-R	CATCTAATTCAACAAGAATTGGGAC
NoYUC8-F	ATGGGTAAATCAACGTTTGAG
NoYUC8-R	TTAGAACTGTTGAGAGATATA
NoAMI1-F	TGCGTTCCCTCTCTTATGGC
NoAMI1-R	ATAGCTTCCTGGATCCGTGC
NoYUC1-F	TTGCCACTGGTTACAAAAGCA
NoYUC1-R	CGTTTTGGGCATCCCTTCTT
NoYUC3-F	CCAAAACTCGATTCCCGACG
NoYUC3-R	CCTTCACGTTTCAAGCCTGC
NoNIT1-F	TTGAGGAAGCGGCGAAAGAT
NoNIT1-R	TGCGAAACTCATCACGACCA
NoNIT2-F	ATTCACCAATGGCGAGCCC
NoNIT2-R	TGTAACTCTTGCTCGGCGAT
NoIAR3-F	AGCGTCCATCTCTTCCCAAC
NoIAR3-R	GACCATGCACCGTCACTGTA
NoILR1-F	GGAGCTAGCCATGGAGGTTG
NoILR1-R	GGTCAGCCTAGAAGACGGTG
NoATMES16-F	TTACACTCTGGCCACCACAC
NoATMES16-R	GTCAGTCTCTTCAGCTTTGGG
NoATMES18-F	ATCAGCACGGATCCAGGAAG
NoATMES18-R	CCCCCGACCAAAGTTGAGAG

A

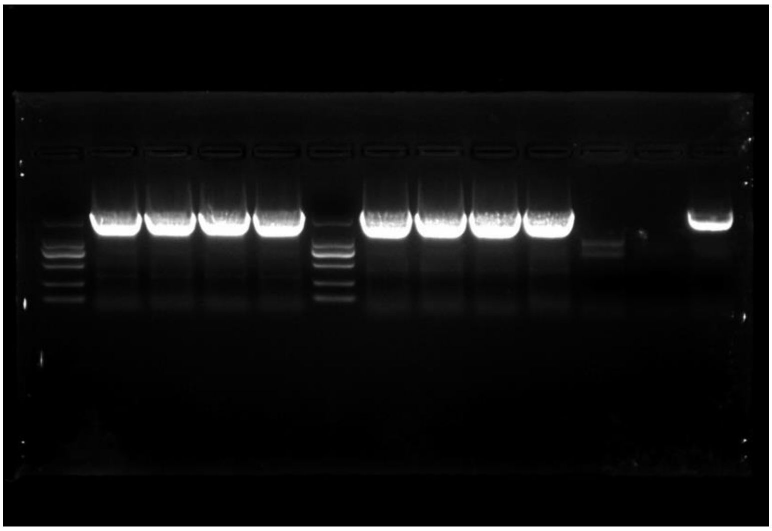

B

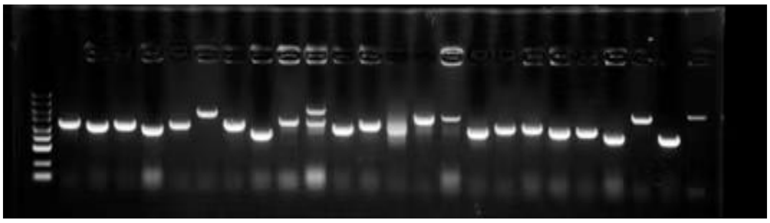

Fig.S1 The PCR test results of *35::NoYUC8* (A) and *DR5::EGFP* (B) transgenic watercress

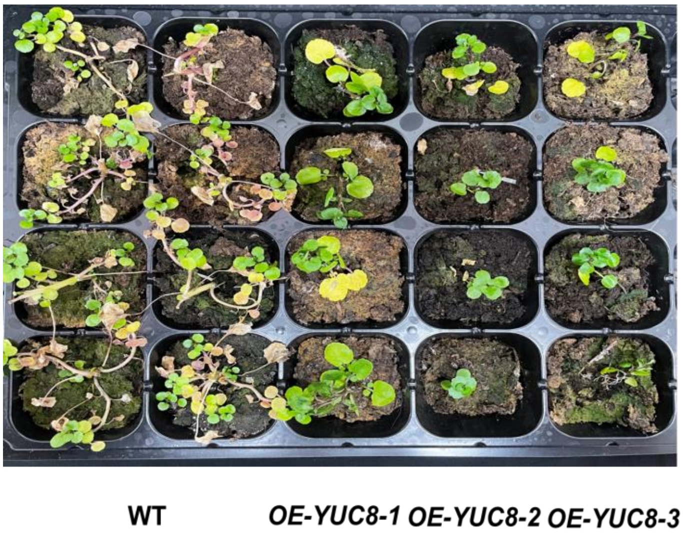

Fig.S2 Effect of *NoYUC8* gene overexpression on watercress cultivated on land

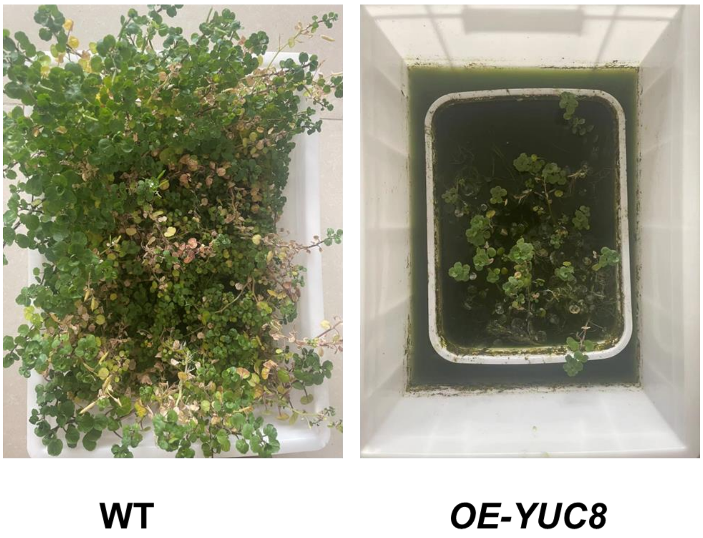

Fig.S3 Effect of *NoYUC8* gene overexpression on watercress

Steps of genetic transformation of watercress

1. The aseptic seedlings of watercress were selected and the leaves and bud primordia were removed.
2. The remaining stem segments were cut and inoculated into MS medium (PH 5.2, add 0.6 M mannitol + 4 mg/L 6-BA). Put it in the light culture box and pretreat it for one day under the condition of 24℃ and light for 16 hours.
3. The OD of bacterial liquid was adjusted to 0.2-0.3 before infection.
4. Put the pretreated stem into the syringe, draw the bacterial liquid and seal for 3 minutes for negative pressure infection.
5. The stem segments were inoculated into MS medium (pH 5.2, add 4 mg/L 6-BA +1.5 mg/L TDZ +1.5 mg/L 2,4-D + 50mg/L Inositol +10mg/L AS) after infection. Put it in the light culture box for three days under the condition of 24℃ and light for 0 hours.
6. After culturing, the stem segments were washed with sterile water, and then inoculated into MS medium (pH 5.2, add 4 mg/L 6-BA + 1.5 mg/L TDZ + 1.5 mg/L 2,4-D + 50mg/L Inositol + 200mg/L Cef + 100mg/L Timentin). After cultured in 24℃ for 16 h for 15 days, callus was induced and GFP fluorescence was observed.
7. The callus was inoculated into MS medium（pH 5.2, add 6-BA 3 mg/L+TDZ 3 mg/L+ Kanamycin 200mg/L). Then the callus was cultured in light culture box for 15 days (the conditions are the same as above) to induce callus to differentiate into buds.
8. The resistant buds were inoculated into MS medium (add Kanamycin 200mg/L). Then cultured in light culture box for 7 days (same as above), rooting was induced and GFP fluorescence was observed.
9. The tissue culture bottle cap was half-opened and half-closed, and the regenerated plants of watercress were domesticated in a light culture box. After 3 days, the regenerated plants of watercress were washed and transferred to outdoor cultivation for PCR detection.
